# IUC26674-90 Impact of delayed radical cystectomy following neoadjuvant chemotherapy on oncological outcomes in Muscle-invasive bladder cancer

**DOI:** 10.1093/oncolo/oyag286.007

**Published:** 2026-08-21

**Authors:** J Bondjeke Nkumindamu, L Afani, O Zouiten, M El Fadli, R Belbaraka

**Affiliations:** Medical oncology department, Mohammed VI University Hospital, Marrakesh - Morocco; Medical oncology department, Mohammed VI University Hospital, Marrakesh - Morocco; Medical oncology department, Mohammed VI University Hospital, Marrakesh - Morocco; Medical oncology department, Mohammed VI University Hospital, Marrakesh - Morocco; Medical oncology department, Mohammed VI University Hospital, Marrakesh - Morocco

## Abstract

**Background:**

Radical cystectomy (RC) following neoadjuvant chemotherapy (NAC) remains the standard of care for muscle-invasive bladder cancer (MIBC), yet the optimal interval between completion of NAC and surgery remains debated. A prolonged delay may compromise the oncological benefit of NAC, particularly in resource-limited settings with constrained operating room access.

**Methods:**

This retrospective single-center study included 75 patients with MIBC (cT2-T4a N0-N1 M0) who received platinum-based NAC followed by RC in the medical oncology department of the Mohammed VI University Hospital from January 2020 to December 2023. Patients were stratified into two groups according to the NAC to surgery interval: standard delay (group A:≤8 weeks, n  =  48) versus prolonged delay (group B:>8 weeks, n  =  27). No patient received adjuvant immunotherapy. Endpoints were pathological complete response (ypT0N0), recurrence-free survival (RFS), and overall survival (OS), analyzed using Kaplan-Meier estimates and Cox regression modeling.

**Results:**

Median age was 66 years (range: 58-77), with a male-to-female ratio of 4:1. Hematuria was the main reason for consultation (71.4%), followed by irritative/obstructive urinary symptoms (28.6%); 51.2% had tobacco exposure. Chemotherapy regimens included cisplatin-gemcitabine (90.4%) and carboplatin-gemcitabine (9.6%, cisplatin ineligible patients). Median NAC to surgery interval was 6.8 weeks in group A versus 13.4 weeks in group B. Pathological complete response (ypT0N0) occurred in 31% (n  =  15) of group A versus 15 % (n  =  4) of group B (p  =  0.04). Two-year RFS was 68% in group A versus 44% in group B, respectively (HR:2.1; 95% Cl:1.1-4.0; p  =  0.02). Two years OS was 79% in group A versus 58 % in group B, respectively (HR:1.9; 95% Cl:1.0-3.7; p  =  0.05). On multivariate analysis, delay > 8 weeks remained independently associated with increased recurrence risk (adjusted HR:1.9; 95% Cl:1.0-3.6; p  =  0.04) after adjustment for clinical stage and ECOG PS.

**Conclusions:**

A cystectomy delay exceeding 8 weeks after NAC was associated with significantly lower pathological complete response, RFS, and OS. These findings highlight the critical need to optimize surgical pathways and resource allocation to ensure timely management of MIBC.

